# Supplementary figures and images for: ACE2 and TMPRSS2 distribution in the respiratory tract of different animal species and its correlation with SARS-CoV-2 tissue tropism
Source: Microbiol Spectr. 2024 Jan 17;12(2):e03270-23. doi: 10.1128/spectrum.03270-23 (PMC10846196; doi:10.1128/spectrum.03270-23)

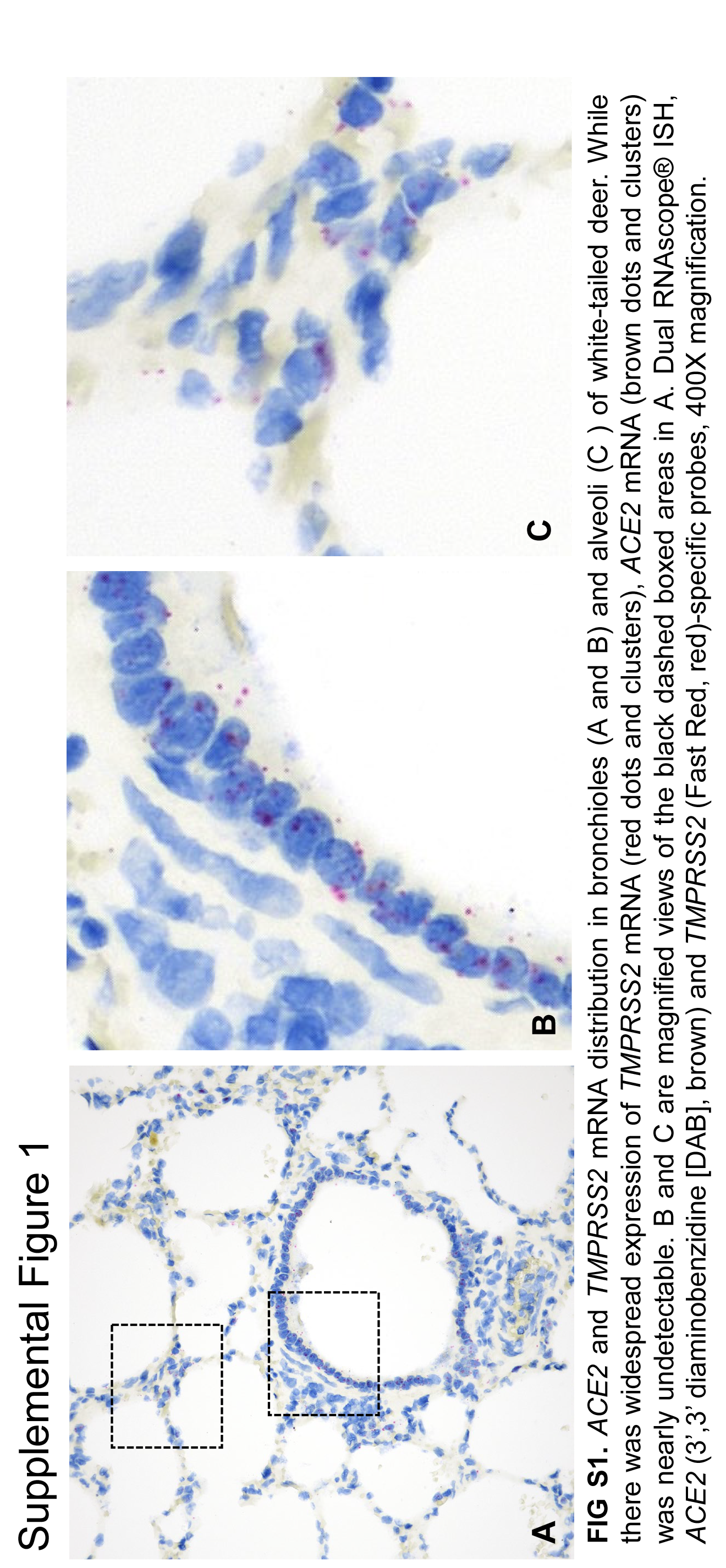

Supplement: Fig. S1 — ACE2 and TMPRSS2 mRNA distribution in bronchioles and alveoli of white-tailed deer. [file spectrum.03270-23-s0001.tif]

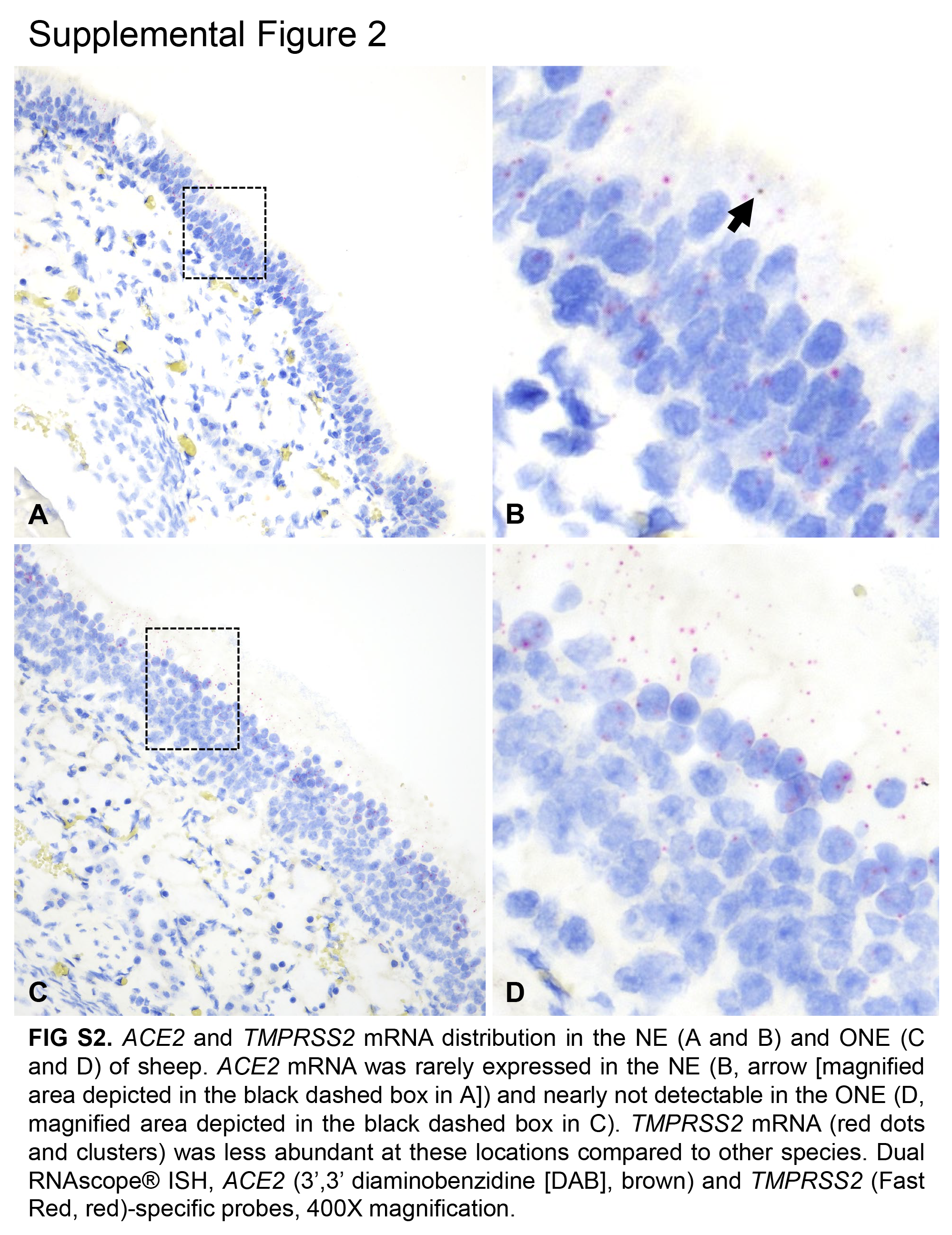

Supplement: Fig. S2 — ACE2 and TMPRSS2 mRNA distribution in the NE and ONE of sheep. [file spectrum.03270-23-s0002.tif]

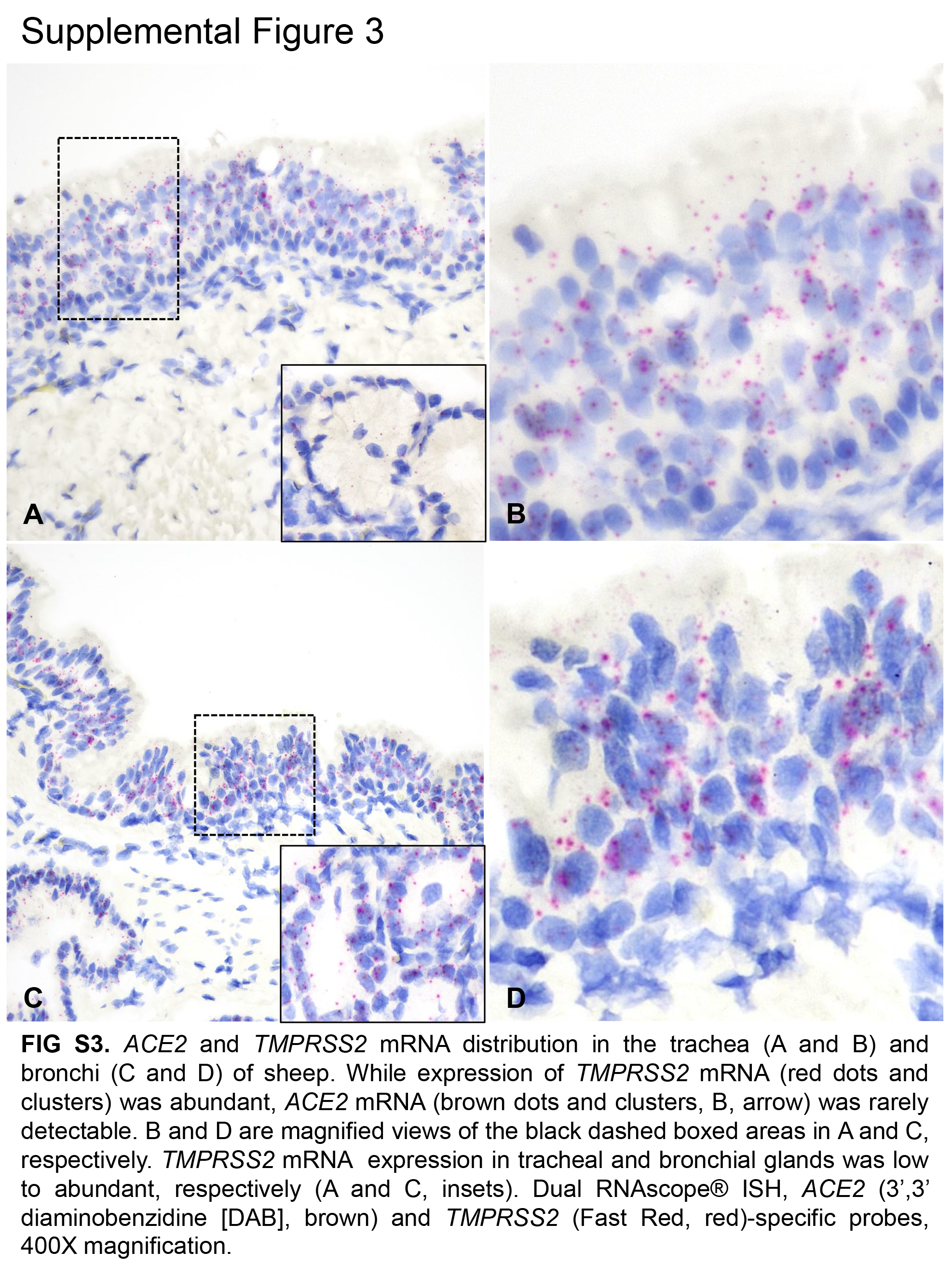

Supplement: Fig. S3 — ACE2 and TMPRSS2 mRNA distribution in the trachea and bronchi of sheep. [file spectrum.03270-23-s0003.tif]

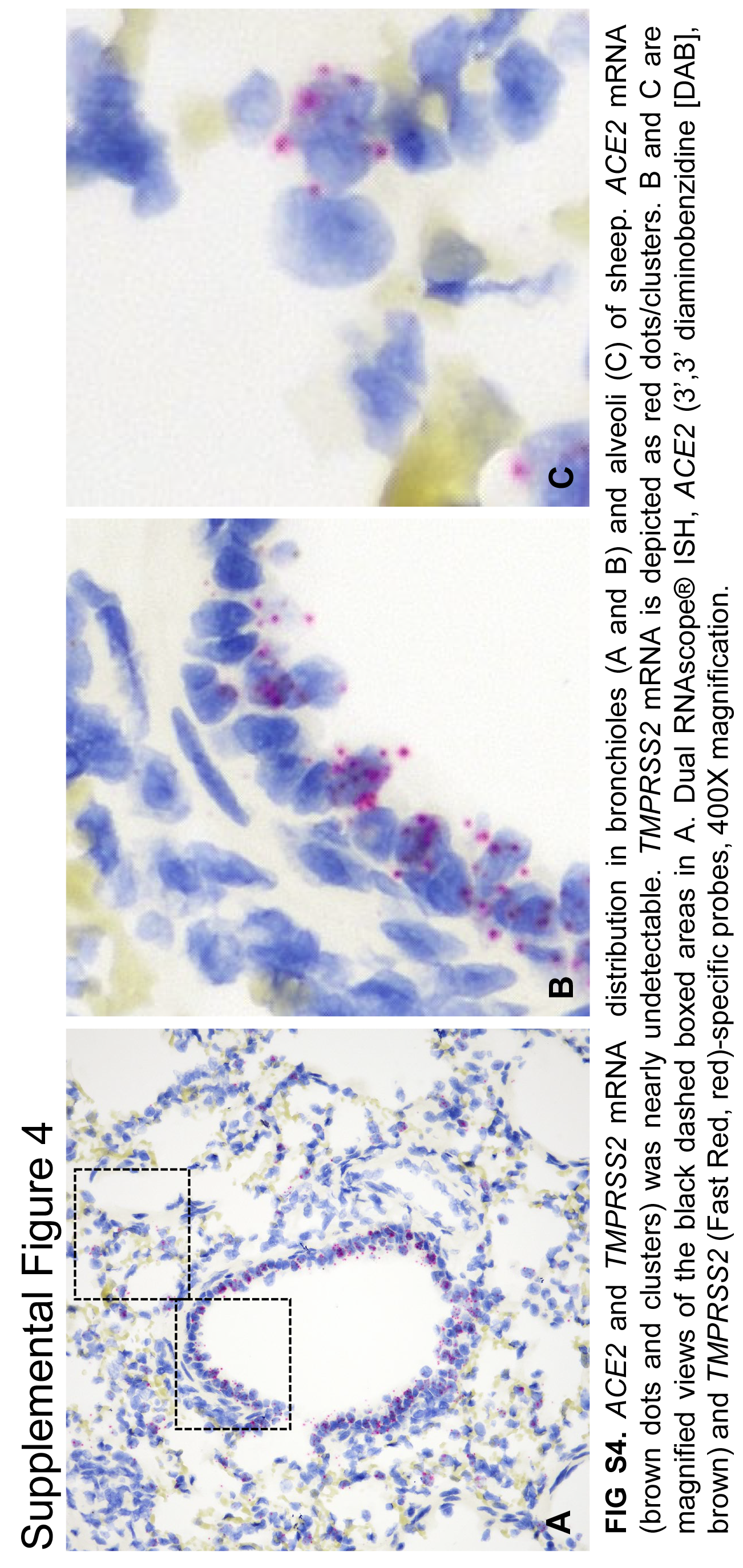

Supplement: Fig. S4 — ACE2 and TMPRSS2 mRNA distribution in bronchioles and alveoli of sheep. [file spectrum.03270-23-s0004.tif]

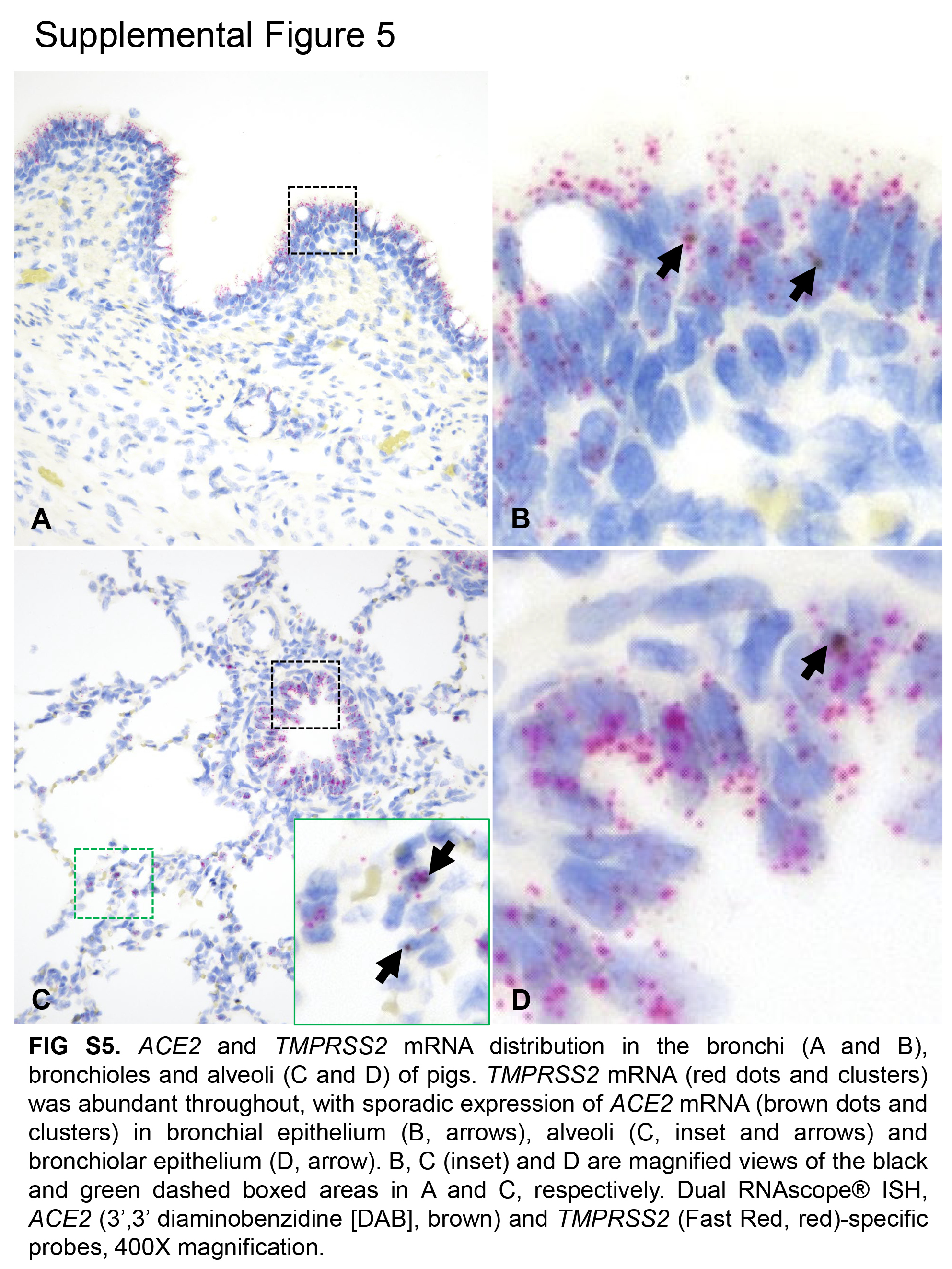

Supplement: Fig. S5 — ACE2 and TMPRSS2 mRNA distribution in bronchi, bronchioles, and alveoli of pigs. [file spectrum.03270-23-s0005.tif]

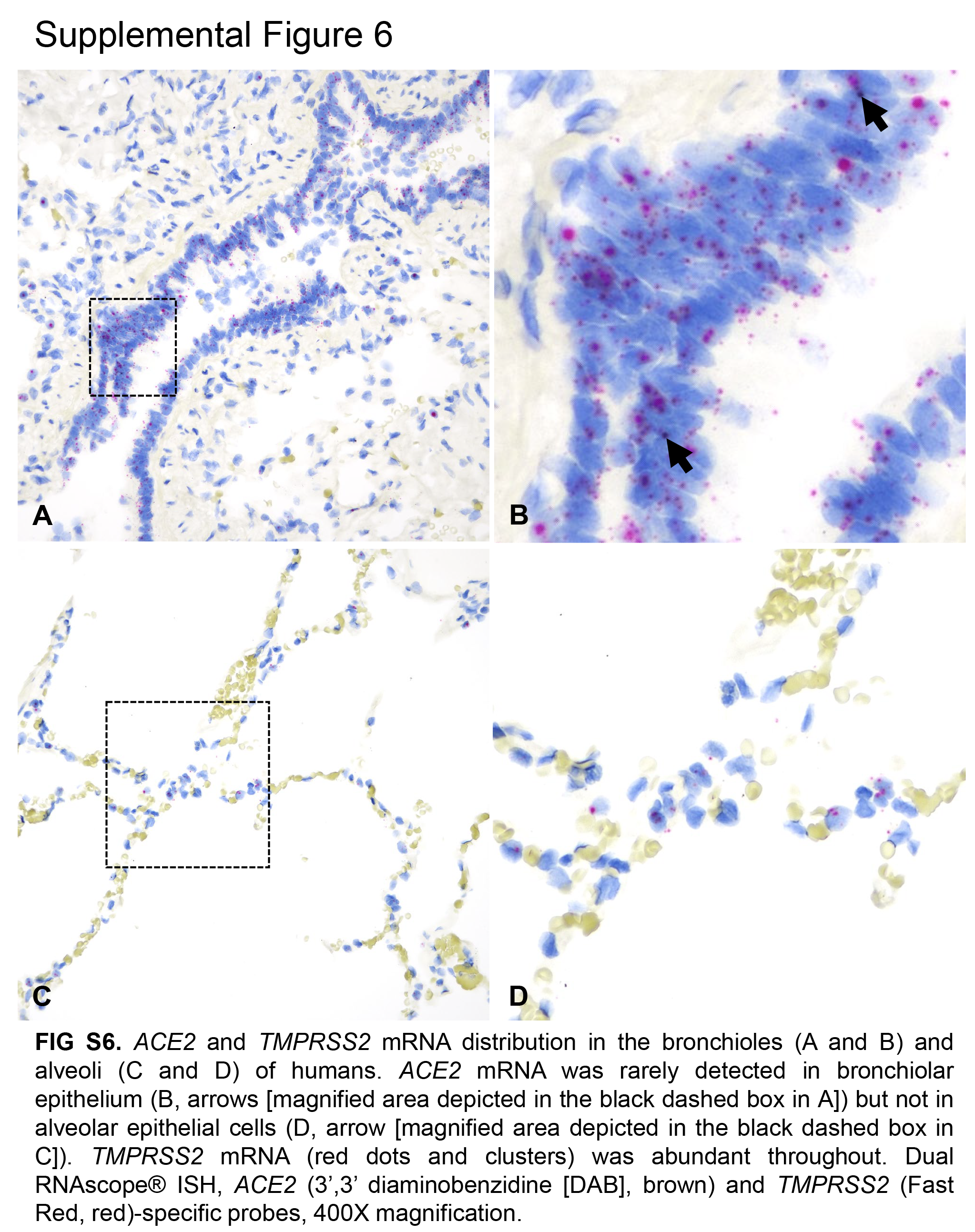

Supplement: Fig. S6 — ACE2 and TMPRSS2 mRNA distribution in bronchioles and alveoli of humans. [file spectrum.03270-23-s0006.tif]

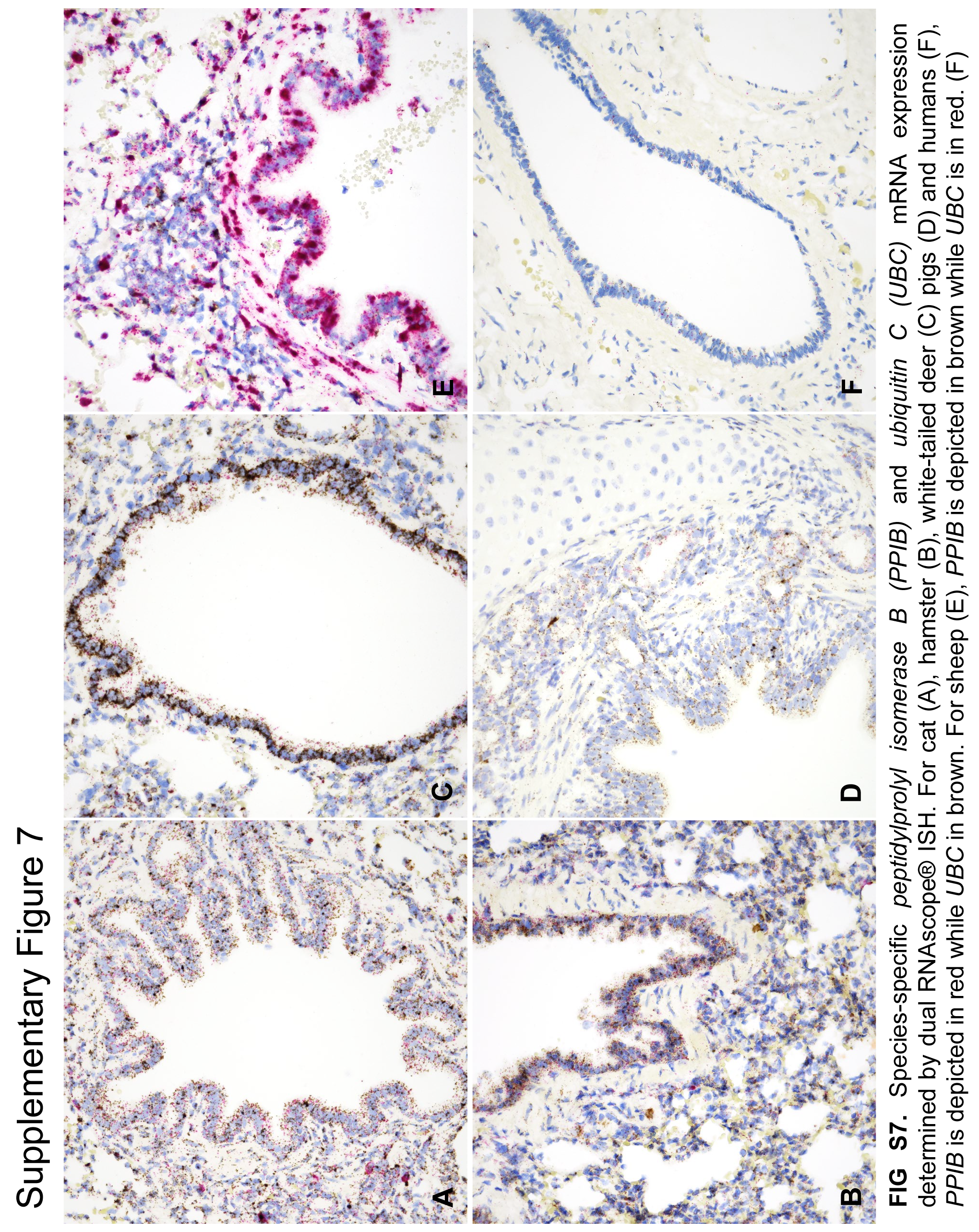

Supplement: Fig. S7 — Species-specific peptidylprolyl isomerase B (PPIB) and ubiquitin C (UBC) mRNA expression. [file spectrum.03270-23-s0007.tif]

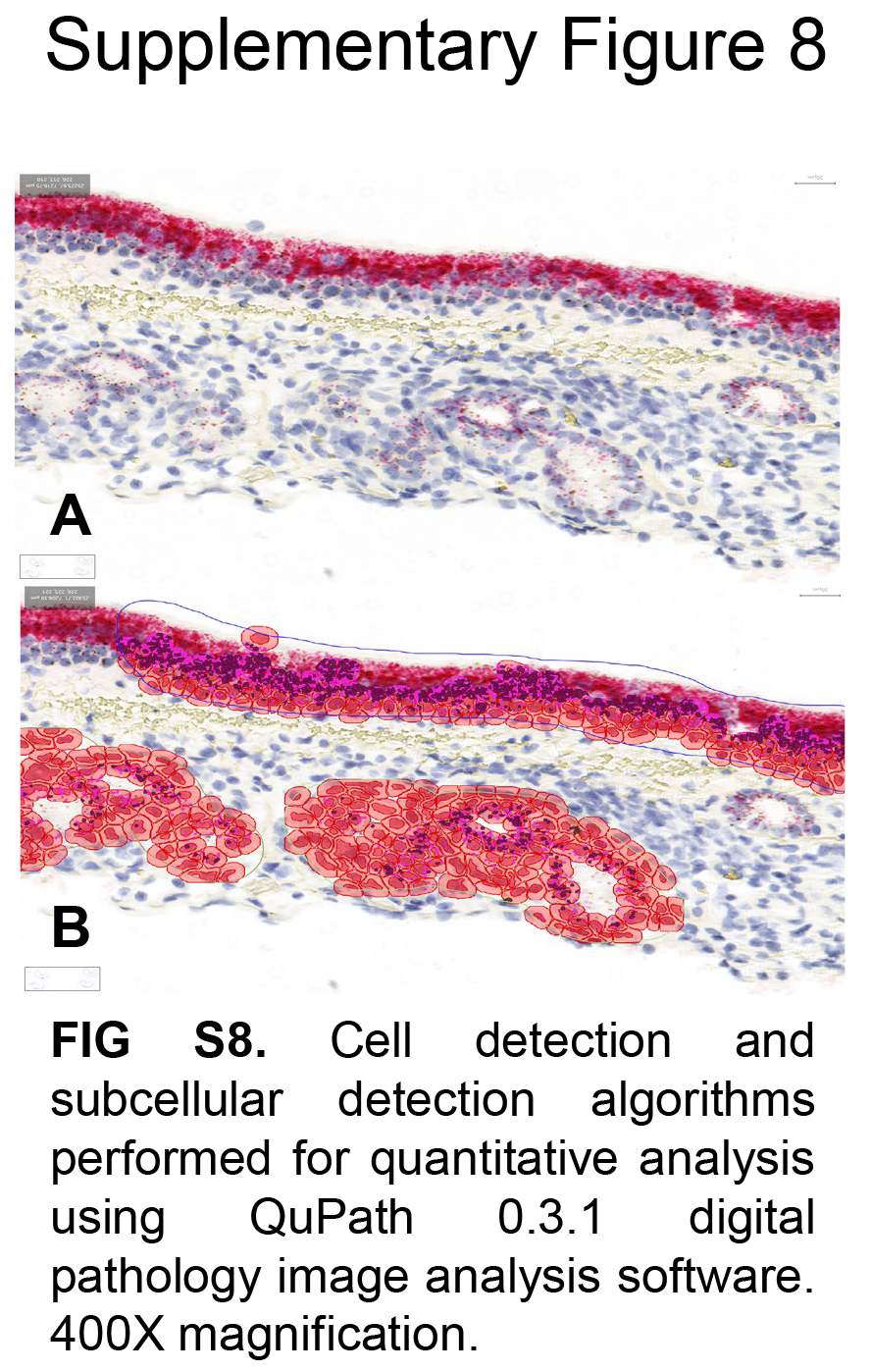

Supplement: Fig. S8 — Cell detection and subcellular detection algorithms performed for quantitative analysis. [file spectrum.03270-23-s0008.tif]
