# Supplementary material for: ACE2 and TMPRSS2 distribution in the respiratory tract of different animal species and its correlation with SARS-CoV-2 tissue tropism
Source: Microbiol Spectr. 2024 Jan 17;12(2):e03270-23. doi: 10.1128/spectrum.03270-23 (PMC10846196; doi:10.1128/spectrum.03270-23)
Supplement: Table S1 — P-values corresponding to the multiple comparisons performed to determine differences in ACE2 and TMPRSS2 mRNA abundance between tissues. [file spectrum.03270-23-s0009.docx]

**Supplementary Table 1.** p-values corresponding to the multiple comparisons performed to determine differences in *ACE2* and *TMPRSS2* mRNA abundance between tissues. Tukey’s post-hoc test, p-values<0.05 were considered significant and are marked with an *. (A) Feline *ACE2*, (B) Feline *TMPRSS2*, (C) Hamster *ACE2*, (D) Hamster *TMPRSS2*, ( E) White-tailed deer *ACE2*, (F) White-tailed deer *TMPRSS2*, (G) Sheep *ACE2*, (H) Sheep *TMPRSS2*, (I) Swine *ACE2*, (J) Swine *TMPRSS2*, (K) Human *ACE2*, (L) Human *TMPRSS2* mRNA

| 1. **Feline *ACE2*** |  |
| --- | --- |
| **Comparison** | **p-Value** |
| Tracheal gland vs ONE | 0.0545 |
| Tracheal gland vs Alveoli | 0.0423* |
| Bronchi vs ONE | 0.2000 |
| Trachea vs ONE | 0.3529 |
| Bronchi vs Alveoli | 0.1841 |
| Tracheal gland vs Bronchioles | 0.2625 |
| Trachea vs Alveoli | 0.3655 |
| Bronchial gland vs ONE | 0.5576 |
| NE vs ONE | 0.6870 |
| Tracheal gland vs NE | 0.7092 |
| Bronchial gland vs Alveoli | 0.5962 |
| Tracheal gland vs Bronchial gland | 0.7390 |
| NE vs Alveoli | 0.7463 |
| Bronchi vs Bronchioles | 0.7016 |
| Trachea vs Bronchioles | 0.8835 |
| Bronchioles vs ONE | 0.9400 |
| Tracheal gland vs Trachea | 0.9589 |
| Bronchioles vs Alveoli | 0.9754 |
| Bronchi vs NE | 0.9843 |
| Tracheal gland vs Bronchi | 0.9872 |
| Bronchial gland vs Bronchioles | 0.9856 |
| Bronchi vs Bronchial gland | 0.9919 |
| NE vs Bronchioles | 0.9967 |
| Trachea vs NE | 0.9986 |
| Trachea vs Bronchial gland | 0.9996 |
| Alveoli vs ONE | 1.0000 |
| Bronchi vs Trachea | 1.0000 |
| Bronchial gland vs NE | 1.0000 |

| 1. **Feline *TMPRSS2*** |  |
| --- | --- |
| **Comparison** | **p-Value** |
| Bronchi vs Alveoli | <.0001* |
| Bronchi vs ONE | 0.0002* |
| Trachea vs Alveoli | 0.0001* |
| Bronchi vs Bronchial gland | <.0001* |
| Bronchi vs Tracheal gland | 0.0003* |
| NE vs Alveoli | 0.0006* |
| Bronchioles vs Alveoli | 0.0014* |
| Trachea vs ONE | 0.0388* |
| Bronchi vs Bronchioles | 0.0250* |
| Trachea vs Bronchial gland | 0.0414* |
| NE vs ONE | 0.1162 |
| Trachea vs Tracheal gland | 0.1089 |
| Bronchi vs NE | 0.1388 |
| NE vs Bronchial gland | 0.1412 |
| Bronchioles vs ONE | 0.2466 |
| Tracheal gland vs Alveoli | 0.2119 |
| NE vs Tracheal gland | 0.2975 |
| Bronchial gland vs Alveoli | 0.2752 |
| Bronchi vs Trachea | 0.3753 |
| Bronchioles vs Bronchial gland | 0.3127 |
| Bronchioles vs Tracheal gland | 0.5564 |
| ONE vs Alveoli | 0.7277 |
| Trachea vs Bronchioles | 0.9356 |
| Tracheal gland vs ONE | 0.9965 |
| NE vs Bronchioles | 0.9988 |
| Trachea vs NE | 0.9992 |
| Bronchial gland vs ONE | 0.9998 |
| Tracheal gland vs Bronchial gland | 1.0000 |

| 1. **Hamster *ACE2*** |  |
| --- | --- |
| **Comparison** | **p-Value** |
| Bronchi vs ONE | 0.0097* |
| Bronchi vs Alveoli | 0.0073* |
| Bronchioles vs ONE | 0.0064* |
| Bronchioles vs Alveoli | 0.0037* |
| Bronchi vs Bronchioles | 0.9881 |
| Alveoli vs ONE | 0.9975 |

| 1. **Hamster *TMPRSS2*** |  |
| --- | --- |
| **Comparison** | **p-Value** |
| Bronchi vs Alveoli | 0.0029* |
| Bronchioles vs Alveoli | 0.0015* |
| ONE vs Alveoli | 0.2116 |
| Bronchi vs ONE | 0.3695 |
| Bronchioles vs ONE | 0.3321 |
| Bronchi vs Bronchioles | 1.0000 |

| 1. **White-tailed deer *ACE2*** |  |
| --- | --- |
| **Comparison** | **p-Value** |
| Bronchi vs Alveoli | 0.0013* |
| Bronchioles vs Alveoli | 0.0081* |
| NE vs Alveoli | 0.0943 |
| Trachea vs Alveoli | 0.2310 |
| Bronchi vs ONE | 0.2113 |
| ONE vs Alveoli | 0.3165 |
| Bronchi vs Trachea | 0.4837 |
| Bronchi vs NE | 0.4033 |
| Bronchioles vs ONE | 0.5970 |
| Bronchioles vs Trachea | 0.8706 |
| Bronchioles vs NE | 0.8557 |
| Bronchi vs Bronchioles | 0.9645 |
| NE vs ONE | 0.9940 |
| Trachea vs ONE | 0.9991 |
| NE vs Trachea | 1.0000 |

| 1. **White-tailed deer *TMPRSS2*** |  |
| --- | --- |
| **Comparison** | **p-Value** |
| Bronchioles vs Alveoli | <.0001* |
| Bronchioles vs ONE | 0.0003* |
| Bronchi vs Alveoli | 0.0003* |
| Bronchi vs ONE | 0.0014* |
| Trachea vs Alveoli | 0.0046* |
| Trachea vs ONE | 0.0154* |
| NE vs Alveoli | 0.0214* |
| NE vs ONE | 0.0759 |
| Bronchioles vs NE | 0.1307 |
| Bronchi vs NE | 0.4230 |
| Trachea vs NE | 0.8514 |
| Bronchioles vs Trachea | 0.8635 |
| Bronchioles vs Bronchi | 0.9758 |
| Bronchi vs Trachea | 0.9957 |
| ONE vs Alveoli | 0.9984 |

| 1. **Sheep *ACE2*** |  |
| --- | --- |
| **Comparison** | **p-Value** |
| ONE vs Alveoli | 0.3207 |
| NE vs Alveoli | 0.0375* |
| Tracheal gland vs Alveoli | 0.0737 |
| Trachea vs Alveoli | 0.1022 |
| ONE vs Bronchioles | 0.8638 |
| ONE vs Bronchi | 0.8790 |
| ONE vs Bronchial gland | 0.9020 |
| NE vs Bronchioles | 0.5340 |
| NE vs Bronchi | 0.5696 |
| NE vs Bronchial gland | 0.6292 |
| Bronchial gland vs Alveoli | 0.6901 |
| Tracheal gland vs Bronchioles | 0.7318 |
| Bronchi vs Alveoli | 0.7463 |
| Tracheal gland vs Bronchi | 0.7644 |
| Bronchioles vs Alveoli | 0.7782 |
| Tracheal gland vs Bronchial gland | 0.8150 |
| Trachea vs Bronchioles | 0.8206 |
| Trachea vs Bronchi | 0.8479 |
| Trachea vs Bronchial gland | 0.8882 |
| ONE vs Trachea | 0.9998 |
| ONE vs Tracheal gland | 1.0000 |
| NE vs Trachea | 0.9996 |
| NE vs Tracheal gland | 1.0000 |
| ONE vs NE | 1.0000 |
| Tracheal gland vs Trachea | 1.0000 |
| Bronchial gland vs Bronchioles | 1.0000 |
| Bronchial gland vs Bronchi | 1.0000 |
| Bronchi vs Bronchioles | 1.0000 |

| 1. **Sheep *TMPRSS2*** |  |
| --- | --- |
| **Comparison** | **p-Value** |
| Bronchioles vs Alveoli | 0.3922 |
| Bronchioles vs NE | 0.3983 |
| ONE vs Alveoli | 0.8784 |
| ONE vs NE | 0.8812 |
| Trachea vs Alveoli | 0.4606 |
| Trachea vs NE | 0.4671 |
| Bronchi vs Alveoli | 0.7338 |
| Bronchi vs NE | 0.7401 |
| Bronchial gland vs Alveoli | 0.8116 |
| Bronchial gland vs NE | 0.8171 |
| Tracheal gland vs Alveoli | 0.8198 |
| Tracheal gland vs NE | 0.8252 |
| Bronchioles vs Tracheal gland | 0.9946 |
| Bronchioles vs Bronchial gland | 0.9953 |
| ONE vs Tracheal gland | 0.9999 |
| ONE vs Bronchial gland | 0.9999 |
| Trachea vs Tracheal gland | 0.9982 |
| Trachea vs Bronchial gland | 0.9985 |
| Bronchioles vs Bronchi | 0.9988 |
| ONE vs Bronchi | 1.0000 |
| Trachea vs Bronchi | 0.9998 |
| Bronchi vs Tracheal gland | 1.0000 |
| Bronchi vs Bronchial gland | 1.0000 |
| Bronchioles vs Trachea | 1.0000 |
| Bronchioles vs ONE | 1.0000 |
| ONE vs Trachea | 1.0000 |
| Bronchial gland vs Tracheal gland | 1.0000 |
| NE vs Alveoli | 1.0000 |

| 1. **Swine *ACE2*** |  |
| --- | --- |
| **Comparison** | **p-Value** |
| Trachea vs Alveoli | 0.4085 |
| Bronchi vs Alveoli | 0.5097 |
| Tracheal gland vs Alveoli | 0.7000 |
| Trachea vs Bronchioles | 0.8846 |
| Bronchioles vs Alveoli | 0.9107 |
| Bronchi vs Bronchioles | 0.9424 |
| Tracheal gland vs Bronchioles | 0.9889 |
| Trachea vs Tracheal gland | 0.9939 |
| Bronchi vs Tracheal gland | 0.9992 |
| Trachea vs Bronchi | 0.9998 |

| 1. **Swine *TMPRSS2*** |  |
| --- | --- |
| **Comparison** | **p-Value** |
| Trachea vs Alveoli | <.0001* |
| Bronchi vs Alveoli | 0.0003* |
| Bronchioles vs Alveoli | 0.0009* |
| Tracheal gland vs Alveoli | 0.0041* |
| Trachea vs Tracheal gland | 0.3671 |
| Trachea vs Bronchioles | 0.5606 |
| Trachea vs Bronchi | 0.7880 |
| Bronchi vs Tracheal gland | 0.9352 |
| Bronchioles vs Tracheal gland | 0.9938 |
| Bronchi vs Bronchioles | 0.9949 |

| 1. **Human *ACE2*** |  |
| --- | --- |
| **Comparison** | **p-Value** |
| NE vs Alveoli | 0.0083* |
| Bronchioles vs Alveoli | 0.1491 |
| NE vs Nasal gland | 0.2812 |
| NE vs Trachea | 0.2863 |
| Trachea vs Alveoli | 0.3305 |
| Nasal gland vs Alveoli | 0.4244 |
| NE vs Bronchioles | 0.6475 |
| Bronchioles vs Nasal gland | 0.9619 |
| Bronchioles vs Trachea | 0.9749 |
| Trachea vs Nasal gland | 1.0000 |

| 1. **Human *TMPRSS2*** |  |
| --- | --- |
| **Comparison** | **p-Value** |
| NE vs Alveoli | <.0001* |
| Bronchioles vs Alveoli | <.0001* |
| NE vs Trachea | <.0001* |
| Nasal gland vs Alveoli | 0.0002* |
| Bronchioles vs Trachea | 0.0060* |
| NE vs Nasal gland | 0.0256* |
| Nasal gland vs Trachea | 0.0636 |
| Trachea vs Alveoli | 0.0818 |
| NE vs Bronchioles | 0.1981 |
| Bronchioles vs Nasal gland | 0.8358 |
